# The Role of Computational Models in the Detection of Colorectal Carcinoma and Precancerous Lesions

**DOI:** 10.3390/ijms27177943

**Published:** 2026-09-06

**Authors:** Jelena Zivic, Stefan Jakovljevic, Milos Zivic, Andrija Rancic, Dušan Radojevic, Mladen Maksic, Ilija Ilic, Nikola Milutinovic, Nikola Mirkovic, Stevan Eric, Bojan Stojanovic, Radojica Stolic, Giulio Antonelli, Natasa Zdravkovic

**Affiliations:** 1 Department of Internal Medicine, Faculty of Medical Sciences, University of Kragujevac, 34000 Kragujevac, Serbia; jelena.zivic@fmn.kg.ac.rs (J.Z.); radojevicdusan@yahoo.com (D.R.); asussonicmaster95@gmail.com (M.M.); radojicastolic61@gmail.com (R.S.); natasasilvester@gmail.com (N.Z.); 2 Gastroenterology and Hepatology Clinic, University Clinical Centre of Kragujevac, 34000 Kragujevac, Serbia; 3 Department of Surgery, Faculty of Medical Sciences, University of Kragujevac, 34000 Kragujevac, Serbia; stevan_kg@yahoo.com (S.E.); bojan.stojanovic01@gmail.com (B.S.); 4 Department of General Surgery, University Clinical Center Kragujevac, 34000 Kragujevac, Serbia; 5 Department of Dentistry, Faculty of Medical Sciences, University of Kragujevac, 34000 Kragujevac, Serbia; zivicmilos5@gmail.com; 6 Department of Maxillofacial Surgery, Clinic of Otorhinolaryngology, University Clinical Centre Kragujevac, 34000 Kragujevac, Serbia; 7 Clinic for Gastroenterohepatology, University Clinical Center Niš, 18000 Niš, Serbia; andrija.m.rancic@gmail.com (A.R.); ilijailic.med@gmail.com (I.I.); 8 Department of Digestive Surgery, University Clinical Center Niš, 18000 Niš, Serbia; milutinac88@gmail.com; 9 Department of Vascular Surgery, University Clinical Center Kragujevac, 34000 Kragujevac, Serbia; 10 Department of Anesthesiology, Resuscitation and Intensive Care, University Clinical Center Kragujevac, 34000 Kragujevac, Serbia; 11 Center for Molecular Medicine and Stem Cell Research, Faculty of Medical Sciences, University of Kragujevac, 34000 Kragujevac, Serbia; 12 Nephrology and Dialysis Clinic, University Clinical Centre of Kragujevac, 34000 Kragujevac, Serbia; 13 Department of Biomedical Sciences, Humanitas University, 20072 Pieve Emanuele, Italy; giulio.antonelli@gmail.com; 14 Endoscopy Unit, IRCCS Humanitas Research Hospital, 20089 Rozzano, Italy

**Keywords:** colorectal polyps, colorectal cancer, colonoscopy, artificial intelligence, computer-aided detection, computer-aided diagnosis, adenoma detection rate, adenoma miss rate, quality indicators

## Abstract

Colonoscopy is a key screening method for colorectal cancer (CRC), but its effectiveness is limited. Computer-aided detection (CADe) and computer-aided diagnostics (CADx), as part of an artificial intelligence (AI) system, improve the detection and optical characterization of lesions. This review maps and analyzes the evidence on the application of AI in colonoscopy, with a focus on the detection, segmentation, and characterization of colon neoplasms, available platforms, architectural models and implementation. The review was conducted in accordance with JBI and PRISMA-ScR guidelines, using the PCC framework. Meta-analyses, randomized controlled trials, systematic and narrative reviews, observational studies, guidelines, and consensus documents on the use of AI systems in different phases of colonoscopy were searched. CADe significantly improves adenoma detection and reduces the number of missed lesions. CADx, segmentation, depth of invasion assessment, and detection of learned lesions remain limited and heterogeneous. CADe has strong evidence for improving ADR, whereas current evidence for CADx remains insufficient to support a “resect-and-discard” strategy. Further cost-effectiveness studies are needed. Commercial platforms vary in their features and level of clinical validation. Colonoscopy using AI is a current topic with rapid development. This scoping review comprises heterogeneous literature covering clinical applications, technical aspects, the potential benefits and limitations of AI in improving colonoscopy performance and reducing the burden of colorectal cancer. Further trials involving diverse patient populations across different countries are needed to validate and extend the current evidence.

## 1. Introduction

Colorectal cancer is a malignant neoplasm of the colon and rectum and is one of the most important public health problems of modern times. Due to its high incidence, significant morbidity and mortality, this disease has great medical and socioeconomic importance. Despite advances in diagnosis and treatment, colorectal cancer remains one of the most common malignant diseases in the world, accounting for approximately 10% of all newly diagnosed cancers [1]. The prognosis of the disease largely depends on the stage at diagnosis, with advanced stages having a significantly worse outcome and higher mortality. The disease most often occurs in people over 50 years of age, and the risk of its occurrence increases progressively with the aging of the population. However, in recent years, some regions have seen an increase in the number of patients among younger adults, especially those aged 30 to 50 years [1,2]. According to global estimates for 2022, around two million new cases of colorectal cancer were recorded worldwide, while more than 900,000 people died from the consequences of this disease [3]. The highest incidence of CRC is recorded in Europe, Australia and New Zealand, while the highest mortality rates are recorded in Eastern European countries. In many high-income countries, CRC incidence and mortality have declined in recent years, likely reflecting improved screening programs and earlier detection. Since the outcome of treatment is largely determined by the stage at the time of diagnosis, early detection of the disease plays a key role in improving patient survival [4].

Colorectal cancer is a complex gastrointestinal malignancy arising from the gradual accumulation of genetic, epigenetic, metabolic, and microenvironmental changes that transform normal colonic epithelium into invasive cancer. Rather than representing a single disease, CRC encompasses multiple molecular subtypes with distinct evolutionary trajectories, signaling deregulation, immune profiles, and microbiota interactions [5]. Colorectal cancer development is influenced by multiple lifestyle and environmental factors, including an unhealthy diet, vitamin D deficiency, physical inactivity, obesity, smoking, excessive alcohol consumption, dysbiosis, and Helicobacter pylori infection [6]. Adopting healthy lifestyle habits, reducing exposure to modifiable risk factors and timely recognition of early symptoms can contribute to reducing the burden of this disease, as well as more effective treatment thanks to earlier diagnosis.

Colorectal cancer is often asymptomatic in its early stages, making organized screening essential for early detection and timely treatment. As the disease progresses, symptoms may include changes in bowel habits, rectal bleeding or blood in the stool, abdominal pain, cramps, and bloating. Chronic tumor-related bleeding can cause iron-deficiency anemia, presenting with fatigue, weakness, and pallor [7,8,9]. A retrospective cohort study of 286 patients younger than 50 years with colorectal cancer found that 85% presented with multiple symptoms, most commonly abdominal pain, altered bowel habits, rectal bleeding, and weight loss. More than half had symptoms for over three months before diagnosis, and most tumors were left-sided and diagnosed at an advanced stage [9].

Concurrently, the incidence of colorectal cancer in individuals younger than 50 years has increased over recent decades, highlighting the growing importance of early symptom recognition and timely diagnosis to facilitate early detection and improve clinical outcomes [10,11].

Advances in sequencing, transcriptomics, and multi-omics analyses have significantly advanced our understanding of colorectal carcinogenesis, revealing a complex interplay of genetic instability, immunological alterations, nutrition, microenvironment and tumor biology. In normal crypt epithelium, stem and progenitor cells ensure continuous epithelial renewal, with their proliferation, differentiation, apoptosis and elimination regulated by the Wnt/β-catenin, BMP, Notch, Hedgehog and EGFR signaling pathways [12,13,14]. Disruption of this balance represents one of the early events in tumorigenesis, leading to clonal expansion and gradual replacement of normal crypt architecture with dysplastic cells [15,16]. The classical adenoma–carcinoma model describes the gradual accumulation of multiple driving genetic changes during the transition from normal epithelium through adenoma to invasive adenocarcinoma [17]. However, modern genomic research shows that this process is not strictly linear or uniform, but rather involves multiple overlapping molecular pathways. The most important are chromosomal instability (CIN), microsatellite instability (MSI), and CpG island-associated methylation phenotype change (CIMP), as well as the conventional adenoma–carcinoma pathway, the serrated neoplasia pathway, chronic inflammation-associated carcinogenesis, and hereditary colorectal cancer syndromes [18,19,20]. Different combinations of genetic, epigenetic, and microenvironmental alterations contribute to the pronounced molecular heterogeneity of colorectal cancer, which is why tumors of similar histological structure can show significant differences in biological behavior, prognosis, metastatic potential, and response to therapy.

## 2. Methods

### 2.1. Study Selection Methods

**Figure 1:**
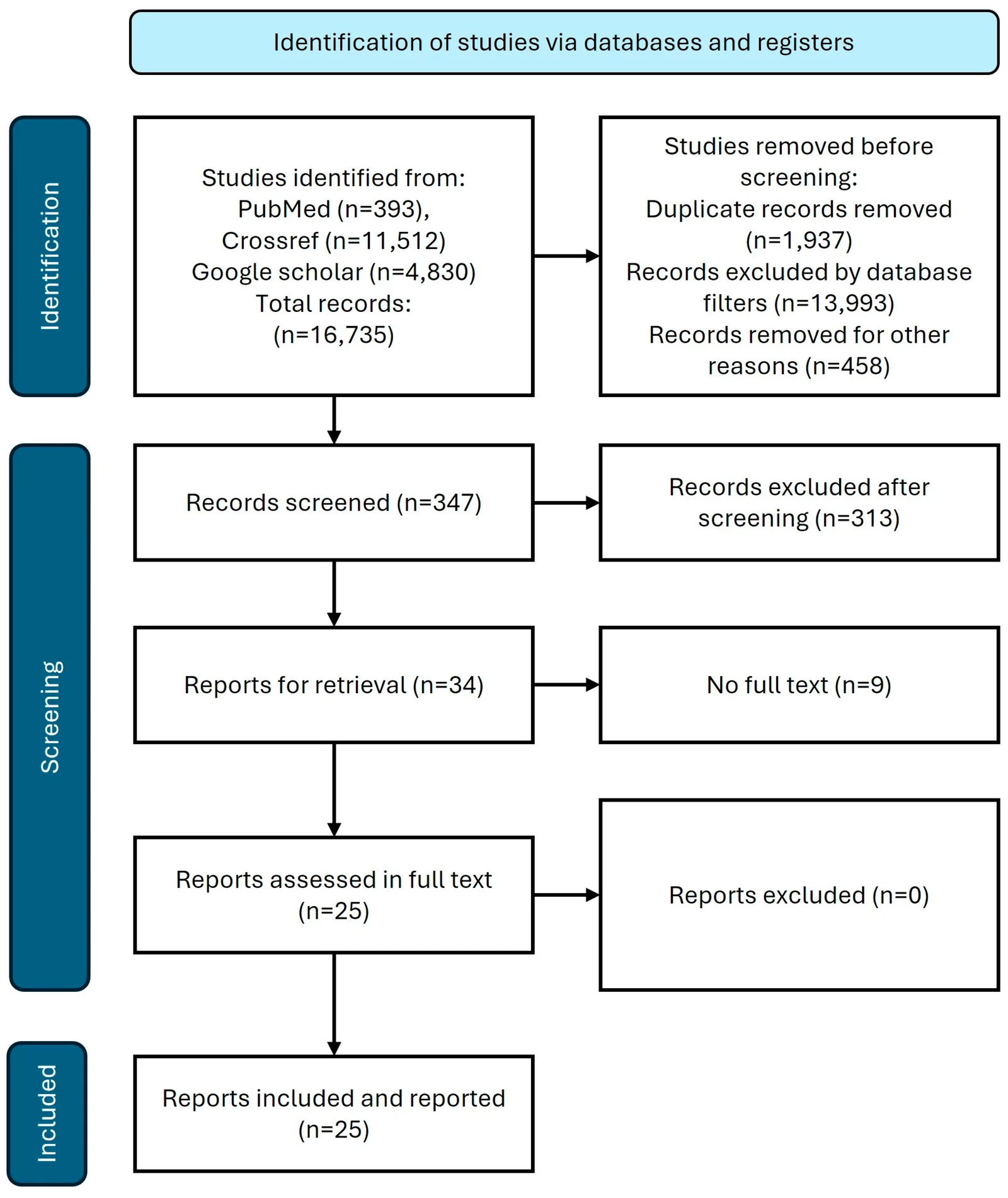
PRISMA flow diagram depicting the search strategy and study selection process.

This scoping review was designed and reported in accordance with the methodological principles of the Joanna Briggs Institute (JBI) guidance for scoping reviews and the Preferred Reporting Items for Systematic Reviews and Meta-Analyses Extension for Scoping Reviews (PRISMA-ScR). The protocol was retrospectively registered on the Open Science Framework (OSF; DOI: https://doi.org/10.17605/OSF.IO/97HZT). The objective was to map and narratively synthesize the current evidence on artificial intelligence (AI)-assisted colonoscopy for colorectal cancer (CRC) screening, surveillance, and early detection, with emphasis on AI-supported lesion detection, optical characterization, invasion-depth assessment, quality indicators, implementation, and commercially available or investigational AI platforms. Because this review used only previously published literature and did not involve human participants, primary data collection, or access to identifiable information, ethical approval was not required (Figure 1).

### 2.2. Eligibility Criteria

Eligibility criteria were defined a priori using the Population–Concept–Context (PCC) framework. The population comprised adults aged 18 years or older undergoing colonoscopy for CRC screening, post-polypectomy surveillance, or early detection of colorectal precancerous lesions. The concept was the application of AI-based or computational systems in colonoscopy, including computer-aided detection (CADe), computer-aided diagnosis or characterization (CADx), computer-aided quality assessment (CAQ), polyp segmentation, invasion-depth assessment, decision-support tools, and autonomous optical-diagnosis approaches. The context included endoscopy units, CRC screening programs, clinical surveillance pathways, and research settings evaluating AI-assisted detection or characterization of colorectal polyps, adenomas, sessile serrated lesions, advanced neoplasia, or CRC.

### 2.3. Inclusion and Exclusion Criteria

Studies were eligible for inclusion if they met the following criteria: (1) evaluated AI, machine learning, deep learning, CADe, CADx, CAQ, segmentation, or related computational approaches; (2) addressed colorectal polyps, adenomas, sessile serrated lesions, advanced neoplasia, CRC, or clinically relevant mucosal lesions; (3) involved colonoscopy or gastrointestinal endoscopy with direct relevance to colorectal lesion detection, characterization, prediction of histology, or procedural quality; (4) reported clinical, technical, diagnostic, or implementation-related outcomes, including adenoma detection rate (ADR), polyp detection rate (PDR), adenoma miss rate (AMR), polyp miss rate (PMR), adenomas per colonoscopy, withdrawal time, optical-diagnosis accuracy, sensitivity, specificity, agreement with histopathology, or quality indicators; and (5) were published in peer-reviewed journals in English. Randomized controlled trials, prospective and retrospective observational studies, comparative studies, technical validation studies, systematic reviews, meta-analyses, narrative reviews, clinical guidelines, and consensus statements were considered when they contributed relevant evidence to the review objectives (Table 1).

Studies were excluded if they focused exclusively on non-colorectal malignancies, pediatric populations, animal or in vitro models, non-endoscopic applications without relevance to colorectal lesion detection or characterization, non-AI endoscopic technologies without a computational component, conference abstracts without sufficient methodological detail, editorials, letters, commentaries, protocols without results, duplicate reports, or publications for which essential methodological or outcome information could not be verified. When multiple reports described overlapping populations or datasets, the most complete and methodologically informative source was prioritized, while related reports were used only for contextual or supplementary information (Table 1).

### 2.4. Information Sources and Search Strategy

A structured literature search was performed in PubMed/MEDLINE for records published from January 2010 to June 2026. Supplementary exploratory searches were conducted in Google Scholar and Crossref to identify additional peer-reviewed publications, guidelines, consensus statements, and relevant technical or implementation studies not captured by the primary PubMed/MEDLINE search. Reference lists of eligible reviews, meta-analyses, guidelines, and key primary studies were also screened manually to reduce the risk of omitting relevant evidence. Web of Science and Scopus were not searched because institutional access was not available.

The search combined controlled vocabulary, where available, with free-text terms related to CRC, colorectal polyps, adenomas, AI, machine learning, deep learning, CADe, CADx, polyp segmentation, colonoscopy, gastrointestinal endoscopy, and quality or implementation outcomes. Boolean operators AND and OR were used to combine terms across the main concepts. The core search terms included “colorectal cancer”, “colorectal neoplasm”, “colorectal adenoma”, “colorectal polyp”, “colon polyp”, “artificial intelligence”, “machine learning”, “deep learning”, “computer-aided detection”, “computer-aided diagnosis”, “CADe”, “CADx”, “polyp segmentation”, “convolutional neural network”, “colonoscopy”, “endoscopy”, “adenoma detection rate”, “adenoma miss rate”, “polyp detection rate”, “polyp miss rate”, “withdrawal time”, and “quality indicators”. The full search strategy and number of retrieved records are summarized in Table 2. Because several AI applications in colonoscopy, particularly CADx, polyp segmentation, invasion-depth assessment, optical diagnosis, and technical-validation studies, may report diagnostic or algorithmic outcomes rather than colonoscopy-quality indicators, supplementary searches were also performed using broader combinations of AI, colorectal lesion, colonoscopy/endoscopy, diagnostic-accuracy, segmentation, validation-metric, and histopathology-related terms without requiring ADR, PDR, AMR, withdrawal time, or quality-indicator terms.

### 2.5. Selection of Sources of Evidence and Data Charting

All retrieved records were exported to Rayyan and reference-management software. Prior to manual screening, automated tools were used strictly for deduplication, to identify and merge duplicate entries across PubMed, Google Scholar, and Crossref. No automated machine-learning filters, AI-based relevance thresholds, or bulk-exclusion tools were used to discard unique records. Consequently, the large proportion of records removed before manual screening reflects the extensive overlap and duplication inherent in combining broad exploratory searches across multiple academic search engines, rather than automated content-based exclusion. Following deduplication, one reviewer screened all unique titles and abstracts against the predefined eligibility criteria, followed by full-text assessment of potentially eligible reports. A second reviewer independently verified all inclusion and exclusion decisions and checked the extracted data for consistency.

Data were charted using a standardized extraction form developed in accordance with the review objectives and the PCC framework. Extracted variables included author, year of publication, clinical setting, study design, population characteristics, sample size, indication for colonoscopy, AI system or platform, developer and software version when reported. Also, we included AI function (CADe, CADx, CAQ, segmentation, combined system, or autonomous optical diagnosis), algorithm architecture, training dataset, validation strategy, comparator or reference standard, outcomes, key findings, false-positive or workflow measures, regulatory status when available, and reported limitations. For CADe studies, specific outcomes included ADR, PDR, AMR, PMR, sessile serrated lesion detection, withdrawal time, and other quality indicators. For CADx and autonomous optical-diagnosis studies, extracted outcomes included sensitivity, specificity, accuracy, area under the curve, positive and negative predictive values, agreement with histopathology, confidence thresholds, non-diagnostic outputs, and concordance with surveillance recommendations. Missing information was recorded as not reported and was not imputed.

Primary clinical and technical studies were used to describe study-specific findings, whereas systematic reviews, meta-analyses, guidelines, consensus statements, and narrative reviews were used to contextualize the evidence base, identify additional relevant sources, and highlight areas of uncertainty. Secondary evidence was not treated as an independent observation when it synthesized primary studies already included in the review. Although the review question was intentionally broad, the number of eligible primary studies was limited because many retrieved records were secondary evidence, technical descriptions, regulatory or commercial documents, conference abstracts, protocols, duplicate reports, or studies not directly evaluating AI-assisted colonoscopy for colorectal lesion detection, characterization, quality assessment, segmentation, or implementation outcomes. Primary clinical and technical studies were therefore synthesized separately from secondary evidence sources. Systematic reviews, meta-analyses, guidelines, consensus statements, and narrative reviews were used only to contextualize the evidence base, identify additional relevant sources, and highlight areas of uncertainty, rather than as independent primary evidence. To reduce the possibility that relevant studies were missed, the PubMed/MEDLINE search was supplemented by exploratory Google Scholar and Crossref searches, manual screening of reference lists from eligible reviews and key primary studies, and independent verification of inclusion decisions by a second reviewer.

Potential overlap was assessed across study characteristics. When identified, the most comprehensive source informed data extraction, while related reports were referenced only for distinct supplementary information.

### 2.6. Synthesis of Results

Findings were synthesized descriptively and narratively according to AI function, clinical purpose, evidence type, platform, validation approach, and reported outcomes. CADe evidence was summarized separately from CADx, CAQ, segmentation, and autonomous optical-diagnosis evidence, because these applications address different clinical tasks and use different performance metrics. Commercially available and investigational AI platforms were mapped according to developer, intended use, training-data transparency, external validation, regulatory status, workflow implications, and key limitations. Owing to heterogeneity in study designs, populations, AI systems, comparators, outcome definitions, and follow-up periods, no quantitative meta-analysis was performed. Consistent with scoping review methodology, formal risk-of-bias assessment was not used as an exclusion criterion; however, major methodological limitations, evidence gaps, and implementation challenges were critically summarized to support interpretation of the mapped evidence.

The present review acknowledges several limitations related to the review process itself. These include the coverage and selection of databases, the comprehensiveness of the search strategy, the inclusion of heterogeneous study designs and clinical settings, the potential overlap of evidence across studies, and the absence of a formal critical appraisal of methodological quality and risk of bias. These factors should be considered when interpreting and generalizing the findings of the present review.

## 3. Colorectal Cancer Screening Tools

Colorectal cancer screening is implemented in many countries, with recommendations varying according to epidemiology, healthcare systems, and available resources. In most European countries, the United Kingdom, and Canada, screening starts at age 50, compared with 45 in the United States, while China determines the starting age according to individual risk [21,22]. According to available data, the average global response to screening programs is about 54%, with pronounced regional differences [23].

**Figure 2:**
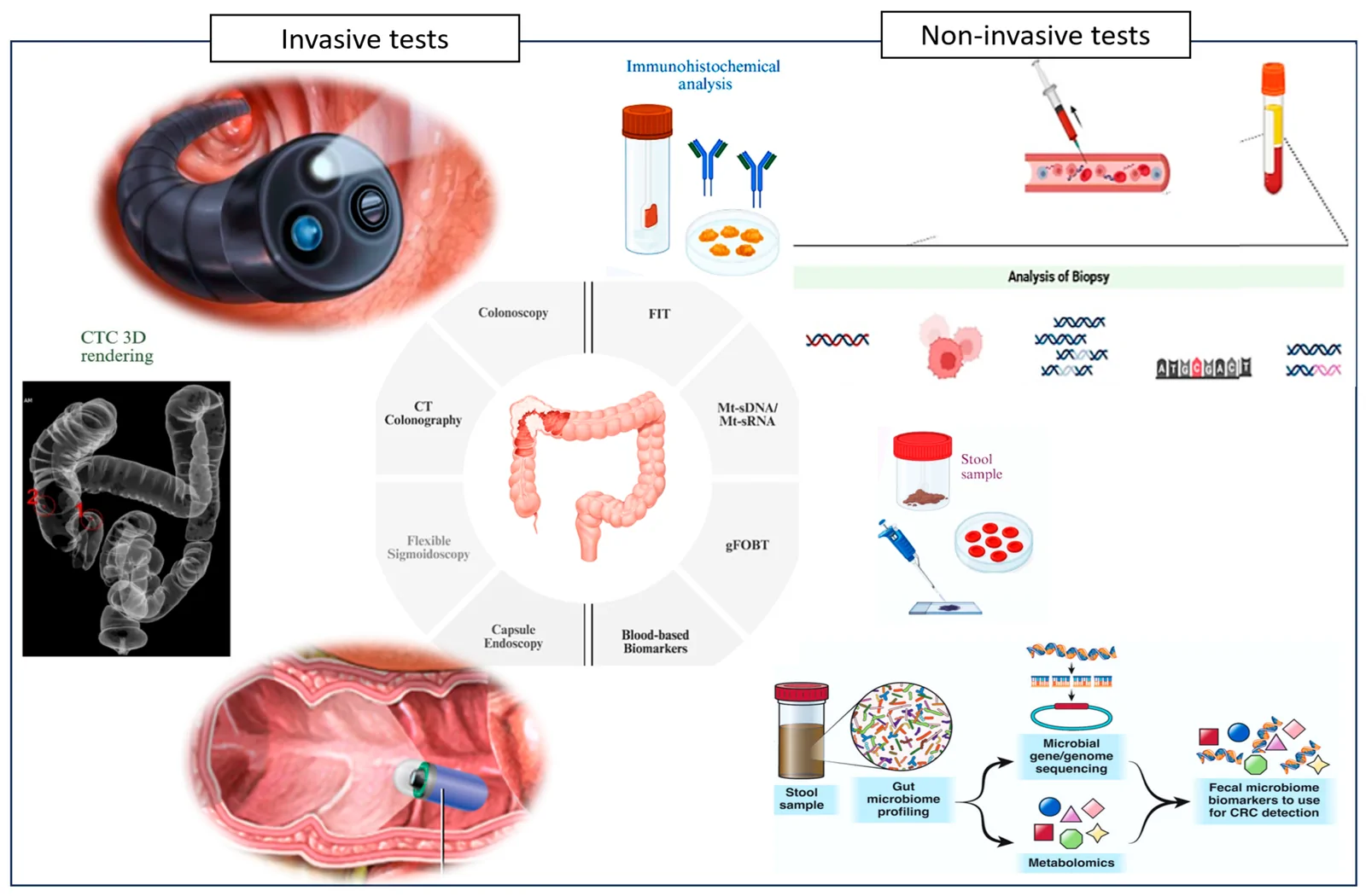
Colorectal cancer screening tests.

Colorectal cancer screening methods are broadly classified as non-invasive or invasive (Figure 2). The American Multidisciplinary Working Group on Colorectal Cancer categorizes these methods according to diagnostic accuracy, cost-effectiveness, and feasibility in routine clinical practice [24]. According to the recommendations of the American Multidisciplinary Task Force on Colorectal Cancer, first-line options include colonoscopy every 10 years and annual fecal immunochemical testing (FIT) [25]. FIT is also recommended by the American Cancer Society and the US Preventive Services Task Force for individuals at average risk. Other non-invasive approaches include stool DNA testing, which detects tumor-associated genetic alterations and is recommended every 3 years in average-risk individuals who are not undergoing colonoscopy [26]. Stool, blood-based, and imaging tests are generally easier to perform and more acceptable to patients than invasive procedures, but some have lower sensitivity or limited cost-effectiveness [22,27]. Colonoscopy and flexible sigmoidoscopy remain established invasive methods because of their high diagnostic accuracy, although their use may be limited by invasiveness, cost, and patient acceptance, particularly in resource-limited settings [22]. Understanding the advantages and limitations of invasive and non-invasive screening methods is essential for selecting the most appropriate strategy based on available resources and healthcare system organization.

### 3.1. Endoscopic Colorectal Cancer Screening Modalities

One meta-analysis of 15,097 patients undergoing colonoscopy reports a remarkable missed detection rate for precancerous polyps: the overall miss rate for adenomas was 26% and for serrated polyps was 27% [28]. Other data indicate that 4% of the patients who previously had colonoscopies for screening or surveillance for cancer that showed normal endoscopic results now get colorectal cancer even after their previous screening [29,30]. These findings, confirmed by other clinical studies and experiences from everyday practice, indicate the limitations of conventional colonoscopy, which are partly due to the variability of the experience and skill of the endoscopist. Consequently, the development of more advanced technologies for the detection of polyps as precancerous lesions has become one of the priorities of modern endoscopy. A growing number of clinical studies confirm that computer-aided detection systems increase the rate of adenoma detection and contribute to a decrease in the number of missed lesions during colonoscopy.

### 3.2. Artificial Intelligence in Colonoscopy

**Figure 3:**
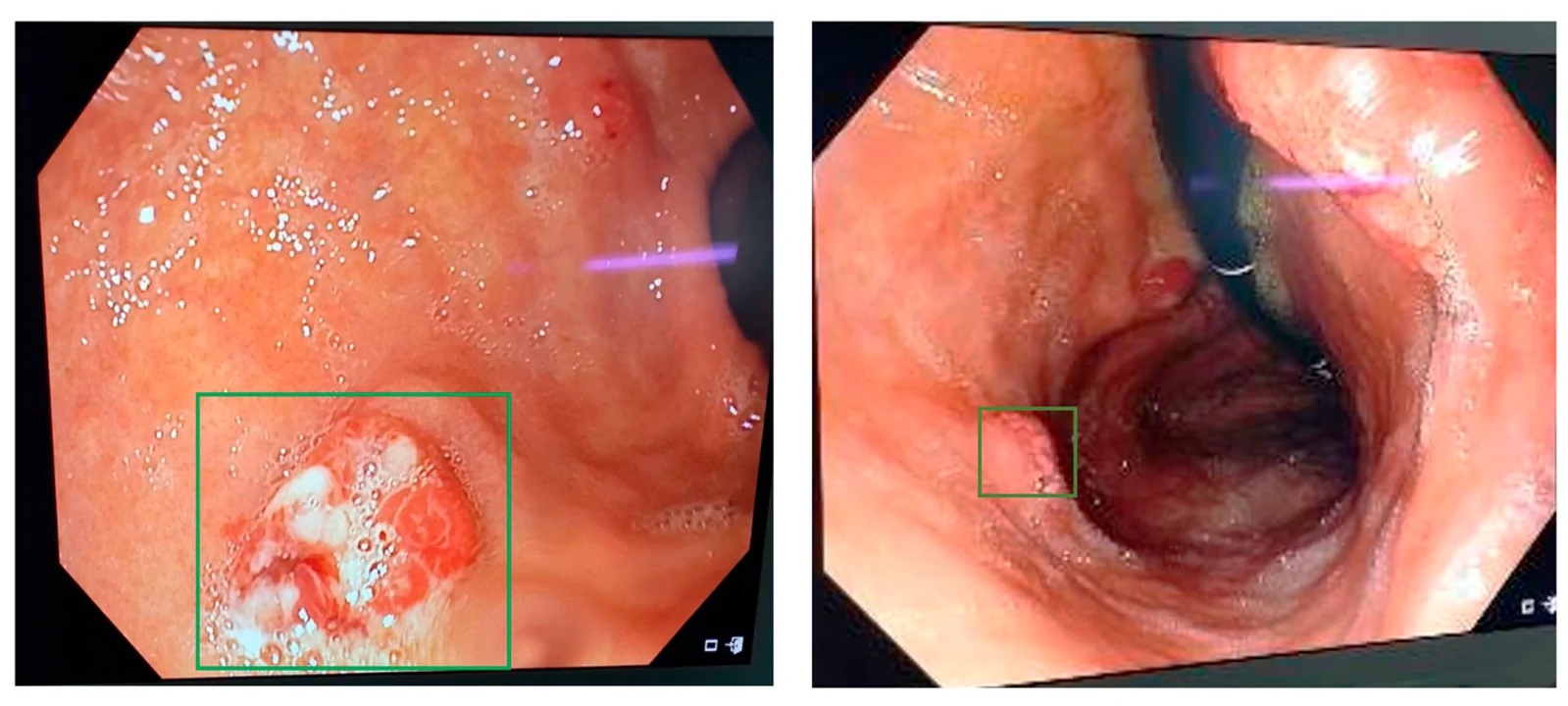
Example of CADe system output during colonoscopy.

Computer-aided diagnostics (CAD) is a broad concept that combines medical image processing, machine and deep learning, computer vision, mathematical and statistical methods, as well as other engineering forensic techniques, and supports doctors in analyzing medical images, detecting pathological changes and making clinical decisions, most often in radiology (Figure 3). CAD systems for colonoscopy are systems for computer-aided analysis of endoscopic images that use artificial intelligence and image processing algorithms for automatic detection and analysis of colon lesions. The systems include real-time video processing, extraction of relevant image features, recognition and localization of suspicious changes, as well as visual marking of lesions to support the endoscopic method in their detection. Based on their functional purpose, CAD systems are traditionally classified into two groups: computer-aided detection (CADe) systems, which identify lesions, and computer-aided diagnosis (CADx) systems, which analyze and characterize them. CADe systems are able to autonomously detect polypoid abnormalities in real time as a patient undergoes colonoscopy, such as in Medtronic’s GI Genius platform, which visually highlights those changes of interest in the video feed presented during the examination. CADe has been reported in clinical studies to yield a ~5% increase in the adenoma detection rate, predominantly in the detection of flat and small polyps that are most frequently missed under routine colonoscopic examination. CADx systems go one step further by analyzing features of polyps using optics to predict whether they will turn out to be adenomatous or hyperplastic—one of which has cancer-forming properties, and the other does not. Therefore, CADx may aid decisions about the excision of endoscopic polyps and possibly decrease unnecessary use of histopathology and costs of treatments [31,32].

### 3.3. Image-Enhanced Endoscopy (IEE) and Chromoendoscopy

Image-enhanced endoscopy (IEE) has provided enhanced differentiation of the mucosal surface pattern and the vascular morphology. This contributes to improved lesion detection and better characterization of the lesion. By using specialized light filters, digital image processing, or contrast dyes, these technologies improve the detection, optical characterization, and precise demarcation of precancerous lesions and early colorectal carcinomas [33]. These technologies include:

- **Virtual chromoendoscopy** uses optical filters or specific light sources to enhance the visualization of the mucosa. The most commonly used technologies are narrow-band imaging (NBI), blue-light imaging (BLI) and linked-color imaging (LCI), which allow for clearer visualization of the surface vascular pattern and mucosal microstructure, thereby improving the detection and characterization of neoplastic lesions [34].
- **Texture and color enhancement imaging (TXI)** is a digital technique that enhances the visualization of tissue texture, surface irregularities, and subtle differences in mucosal color, facilitating the recognition of early neoplastic changes [35].
- **Conventional chromoendoscopy,** texture and color enhancement imaging (TXI), is a digital technique that enhances the visualization of tissue texture, surface irregularities, and subtle differences in mucosal color, facilitating the recognition of early neoplastic changes [36].
- **Chromoendoscopy and Red Dichromatic Imaging—Traditional chromoendoscopy** is based on the direct instillation of contrast dyes, such as methylene blue or indigo carmine, at specific sites of the mucosa to better appreciate crypt architecture, delineate the margins of lesions and more easily identify intra-epithelial neoplastic changes [36,37].
- **Red Dichromatic Imaging (RDI)—**In RDI, light sources are modified in terms of their wavelengths, which then can visualize deeper underlying vasculature and locate bleeding more accurately with the scope [37].

### 3.4. Mechanical Techniques to Improve Lesion Detection

Mechanical devices can complement optical techniques by improving mucosal visualization during colonoscopy. Cap-assisted colonoscopy (CAC) uses a transparent distal cap to flatten mucosal folds and improve visualization of their proximal aspects, while the Endocuff device uses flexible extensions to separate the folds during withdrawal, potentially increasing detection of small and subtle adenomatous lesions [38,39]. Red Dichromatic Imaging (RDI) is a light-filtering technology aiming to visualize deeper intramural vascular structures more easily than conventional white-light colonoscopy, leading to the localization of potential bleeding sites [37]. In addition to optical methods, mechanical devices have been developed to improve visualization of the mucosa during colonoscopy.

### 3.5. Quality Indicators of Colonoscopy

Despite its proven benefits in reducing colorectal cancer incidence and mortality, maintaining high-quality colonoscopy remains challenging. Examination quality is assessed using standardized indicators, including the Boston Bowel Preparation Scale (BBPS), cecal intubation rate (≥95% for screening and surveillance colonoscopies) and ADR. ADR is widely used as a key indicator of colonoscopy quality [40]. For adequate mucosal inspection, it is recommended that the endoscope withdrawal time be no shorter than six minutes [41,42]. It has been found that each 1% increase in ADR is accompanied by a reduction in the risk of interval CRC of approximately 3%, as well as a reduction in mortality of approximately 5% [43]. According to the updated 2024 ASGE/ACG recommendations, the minimum target ADR is ≥35% for patients aged ≥45 years undergoing screening, surveillance, or diagnostic colonoscopy, with sex-specific targets of ≥40% for men and ≥30% for women [44]. In addition to ADR, polyp detection rate (PDR) is used as a supplementary quality indicator. Colonoscopy performance may also vary according to lesion characteristics, anatomical factors, and the endoscopist’s experience and technical skills, contributing to differences in CRC detection rates between operators [45].

To improve the quality of the examination and reduce the missed adenoma rate (AMR), various approaches have been investigated, including optimizing the patient position during colonoscope withdrawal, extending the withdrawal time (WT), using a split dose of bowel preparation, involving an additional observer, and introducing AI-based systems [46,47] (Table 3). In addition to endoscopy, AI algorithms are increasingly used in digital pathology to assess microsatellite instability (MSI) status from digitized histopathological preparations, thereby facilitating identification of patients who require additional molecular testing [46].

## 4. Functional Taxonomy of AI Systems in Colonoscopy

Artificial intelligence is increasingly being used in the early detection of CRC, enabling integrated analysis of clinical data, medical images, and gut microbiome characteristics. Thanks to advanced machine learning algorithms and deep neural networks, it is possible to improve diagnostic accuracy, increase the rate of adenoma detection during colonoscopy, and reduce the impact of inter-endoscopist variability. Results to date show that AI systems achieve better diagnostic performance than conventional approaches in certain clinical scenarios, especially in the detection of early neoplastic changes [47].

Modern AI models are based on various algorithms and technologies, and their application in clinical practice includes the following (Table 4):

CADe and CADx act as a consistent “second reader”. Several studies have shown that the use of CADe technology leads to an increase in ADR and PDR, a decrease in AMR, and a decrease in inter-operator variability, resulting in higher diagnostic accuracy and better quality of colonoscopy examinations [48]. However, the available results of RCTs are not completely uniform, and data on the long-term effects and clinical benefits of the use of CADe systems are still limited [49].

### 4.1. Algorithmic Workflow of CADe Systems

CADe systems performance relies on integrated image analysis, enabling the rapid recognition and localization of suspicious findings during colonoscopy [49]. The first step involves preparation and optimization of input data, during which images are processed to reduce noise, improve contrast and equalize image quality. This procedure provides a more reliable basis for subsequent analysis. The next phase is the extraction of regions of interest, where the algorithm recognizes and isolates relevant anatomical structures from surrounding tissues that are not of diagnostic importance. In this way, the system focuses the analysis on the target organ or lesion and reduces the possibility of misinterpretation [50,51]. The process is called segmentation and, specifically for colorectal polyps, it is the process of automatically recognizing and accurately marking the boundaries of polyps on colonoscopy images [52]. This function is crucial for CADe/CADx, as it helps physicians identify potentially precancerous and malignant changes promptly, while simultaneously reducing the rate of polyps missed during the examination [53]. After initial image analysis, the system identifies and labels potential abnormalities using machine and deep learning algorithms, primarily convolutional neural networks (CNN). Accurate detection of polyps remains a challenge due to the great diversity of their appearance, size, color, and texture, as well as the presence of structures that can mimic polyps during colonoscopy. To improve detection accuracy, CNN models have been developed that simultaneously use convolutional kernels of different sizes, which allows for more efficient feature extraction and more reliable recognition of polyps on colonoscopy images [53]. The algorithm analyzes the morphology, structure, and texture of the tissue to identify atypical patterns that may indicate the presence of polyps or other lesions, and then marks them as suspicious regions [53]. In the final phase, after the algorithm identifies a suspicious change, the system visually marks it on the screen, most often with a colored frame or graphic mark, and activates an audible alert if necessary. In this way, the CADe system increases the likelihood of detecting subtle lesions and reducing the risk of overlooking them [54,55,56]. Each CADe system requires an independent performance assessment before being introduced into clinical practice, which is why the effectiveness is analyzed both at the individual case level and at the level of detected lesions. Quantitative indicators such as the Dice Similarity Coefficient (DSC), the overlap defined by the mean Intersection over Union (IoU) between the automatically determined and reference lesion boundaries, are most often used to assess the accuracy of detection and localization. In addition to technical validation, the clinical benefits of the CADe system are confirmed in prospective, multicenter studies with multiple examiners and a larger number of patients. In this manner, several commercial colonoscopy systems have been evaluated, including the GI Genius™ (Medtronic), CAD EYE (Fujifilm), Endo-AID (Olympus), and Discovery™ (Pentax Medical), with the transmission of colorectal polyp detection. DSC, IoU, and mean distance are the metrics primarily used for detection and segmentation in AI model development, while the clinical performance of commercial systems such as the GI Genius™, CAD EYE, Endo-AID™, and Discovery is evaluated using ADR, PDR, sensitivity and specificity, AMR, false-positive rate, and detection time [57,58,59].

One of the newer CADe systems for colorectal polyp detection during colonoscopy is Magnetic-Colo. It is based on deep learning algorithms and convolutional neural networks, whereby, continuously processing the video stream from the endoscope, the system automatically highlights regions with features suspicious for polyps, thus providing endoscopists with additional support during the examination. In an internal validation, which included 172 colonoscopy videos with 4,656,632 frames and 263 histopathologically confirmed polyps, the system achieved a sensitivity of 99.6% at the polyp level and a specificity of 98%. Previous studies have also shown its compatibility with endoscopic platforms from various leading manufacturers [60].

### 4.2. Basic Principles of CADx Systems

The main difference between CADe and CADx systems is reflected in their functional role. While CADe focuses on detecting suspicious lesions during colonoscopy, CADx is designed to further characterize these findings based on visual features such as morphology, surface, vascular pattern, color, and texture. By assessing these characteristics, CADx can help predict the histological nature of a lesion and support diagnostic decision-making. Thus, CADx complements rather than replaces lesion detection and typically operates on a region of interest identified by the endoscopist or a CADe system. Using deep learning algorithms, these systems estimate the probability that a polyp is neoplastic or non-neoplastic in nature, and the result of the analysis is displayed to the endoscopist through visual indicators and, in some systems, audible signals, thereby providing support in decision-making during colonoscopy. EndoBRAIN is a CADx system that, in combination with high-magnification endocytoscopy, enables automated optical characterization of colorectal polyps in real time. The system analyzes the endoscopic image and displays an assessment of the probability of neoplastic or non-neoplastic nature of the lesion to support the endoscopist [60,61]. The applications of CADx and related algorithms are not limited to medicine, but also include pattern recognition, biometric identification, remote sensing data analysis, and security systems. Early studies have reported promising diagnostic performance of CADx systems, although their effectiveness in routine clinical practice may vary across different settings. This variability may be influenced by factors such as endoscopist experience, lesion morphology, and the quality and characteristics of the data used to train the AI model [60,61].

### 4.3. CADe/x Devices

Integrated CADe/x systems combine real-time lesion detection and characterization during colonoscopy. Following detection, they provide optical assessment to help the endoscopist estimate histological nature and guide clinical decision-making. Their performance is evaluated through technical and clinical validation of detection and characterization accuracy [51].

### 4.4. Artificial Intelligence in Endoscopic Submucosal Dissection

Endoscopic submucosal dissection (ESD) is a minimally invasive, advanced procedure for the removal of early neoplastic and premalignant lesions with larger dimensions from the digestive mucosa. Given that the procedure carries risks, such as bleeding and perforation, AI has been applied to this method. The development and application of AI in ESD have been driven by inadequate transection of submucosal or muscular blood vessels due to the lack of visualization of the depth of resection within the same lesion [62,63].

AI in endoscopic submucosal dissection (ESD) during colonoscopy improves the accuracy of the procedure through CAD. These systems help locate flat lesions, precisely define tumor margins, and distinguish safe tissue layers from high-risk areas [64]. The primary AI functions in ESD in colonoscopy are, first, lesion detection and characterization (CADe/CADx), which locate and mark the margins of lesions to ensure complete resection. The program also estimates the depth of invasion in the colon to make the optimal therapeutic decision (surgery or ESD) (Artificial Intelligence Clinical Decision Support Solution (AI-CDSS)) [65]. Other functions include supporting procedural tasks that facilitate post-procedure reporting, such as identifying safe zones and mapping tissue complications in real time. Considering safety, the potentially faster procedure with fewer unwanted complications is highlighted. The disadvantage of using AI in ESD is the need for a larger number of multicenter studies, as well as the fact that success largely depends on the technique and training of the endoscopist [62].

### 4.5. Computer-Aided Quality Assessment (CAQ)

Alongside CADe and CADx, a third category of artificial intelligence tools has been developed: computer-aided quality assessment (CAQ) systems for colonoscopy. The primary aim of CAQ systems is to assess and monitor colonoscopy quality and to improve the visualization of the colonic mucosa. Their most important functions include monitoring the speed of endoscope withdrawal, as well as recognizing insufficiently examined or unvisualized areas of the mucosa. If the system detects excessive extraction speed or incomplete visualization of certain segments, an appropriate visual warning is displayed to the endoscopist, allowing correction of the examination technique and potentially reducing the risk of missing lesions [66]. CAQ systems provide endoscopists with feedback during the procedure itself, enabling timely correction of technique, easier fulfillment of quality criteria, and gradual improvement of the operator’s practical skills.

## 5. Commercial AI Platforms

Several AI platforms for colonoscopy have already been introduced into clinical practice or are in advanced clinical evaluation. GI Genius is one of the most validated commercial CAD systems for real-time colorectal polyp detection (Table 5). ENDO-AID CADe is available as part of the Olympus endoscopic detection systems, while CAD EYE (Fujifilm) combines detection functions and, in other configurations, polyp characteristics. EndoBRAIN and EndoBRAIN-EYE were primarily developed for optical diagnosis using endoscopy with magnification and enhanced image presentation techniques [67,68]. Other platforms, such as EndoScreener, EndoAngel, Discovery, SKOUT, and EndoMind, are expanding the range of AI solutions available in clinical and research settings [69,70,71]. The capabilities and performance of all these systems vary depending on the algorithm architecture, training datasets, compatibility with endoscopic equipment, and functionality. Independent studies have shown that different CAD systems, as well as different versions of the same software, can achieve different sensitivity and false-positive rates [71]. Table 5 shows the major commercial and near-available AI platforms in colonoscopy, with a focus on their development, training data, external validation, regulatory status, clinical purpose, and limitations. The comparison shows that these platforms are mainly intended to aid in real-time detection of lesions rather than self-diagnosis, while the strength of evidence depends on data transparency, quality of validation, number of false positives, and regulatory approval.

## 6. Impact on Missed Adenomas, Sessile Serrated Lesions and Tandem Study Design

The most clinically relevant finding associated with increased ADR is the concomitant reduction in the miss rate, as demonstrated in both tandem and double-blind studies. CADe-assisted colonoscopy significantly reduced the adenoma miss rate (13.9% vs. 40.0%) and polyp miss rate (13.0% vs. 45.9%) compared with routine white-light colonoscopy (both *p* < 0.0001). The reduction in adenoma miss rate was also significant in the ascending, transverse, and descending colon, suggesting that CADe may improve lesion detection throughout the examined colon [81]. A 2024 meta-analysis demonstrated that AI-assisted colonoscopy significantly improves adenoma detection, regardless of study design, AI system type, or endoscopist experience [80]. Another meta-analysis indicates that AI-assisted endoscopy significantly reduces the miss rates of adenomas, polyps, and sessile serrated lesions, potentially improving the accuracy of GI endoscopic procedures [82]. This implies that some lesions, such as those with a serrated morphology, are harder to “see” for algorithms [83]. Moreover, the tandem study design allows a more direct assessment of missed lesions, although its results may be affected by the endoscopist’s learning process and changes in behavior during the second examination. Therefore, tandem, parallel, and multicenter randomized study designs are commonly used to assess the impact of AI in colonoscopy [84,85] (Table 6 and Table 7).

Where CADe’s output is “identify lesions,” CADx can issue “real-time optical diagnosis and predict histopathology, risk of malignancy, and surveillance intervals”, judged by measures of diagnostic test performance: area under the curve (AUC), sensitivity, specificity, negative predictive value (NPV), and histopathological concordance [77,78,86]. CADx offers the possibility to move towards “resect-and-discard” versus “leave-in situ” decisions for diminutive polyps, thus decreasing pathology workload and saving hospital costs [76,84,87]. Although complex histological lesions may require more sophisticated AI models, autonomous algorithms have the potential to achieve near-perfect diagnostic performance [88]. Rather than replacing endoscopists, their role may be to extend expert-level performance to trainees and community practice [77,78]. As reported in several multicenter trials and side-by-side comparisons, real-time CADe has been associated with increased ADR and PDR and reduced lesion miss rates across different levels of endoscopist expertise, although the magnitude of these effects may vary among studies [89,90,91]. For clinical applicability, AI must achieve an optimal balance between sensitivity and operational burden, including false-positive detections and processing time. Ideally, this should be achieved through a low-latency, endoscopy-integrated system that supports an efficient and ergonomic workflow [92] (Table 6 and Table 7).

**Table 6:** Clinical and technical evidence for CADe systems in AI-assisted colonoscopy.

Study (Year)	Evidence Type/ Setting	Population/Sample	CADe System/Model	Comparator or Reference	Primary Endpoints	Key Quantitative Findings	Validation/ Robustness	False Positives/Workflow	Main Limitations/Bias	Critical Analysis and Synthesis
**Barua et al. (2021)** [64]	Systematic review and meta-analysis of 5 prospective RCTs all conducted in China	4311 participants (2146 AI; 2165 control)	Four CNN-based CADe/quality systems, including EndoScreener and ENDOANGEL	Colonoscopy without AI	ADR, PDR, lesions per colonoscopy, advanced adenomas	ADR 29.6% vs. 19.3%, RR 1.52 (95% CI 1.31–1.77); PDR 45.4% vs. 30.6%, RR 1.48 (1.37–1.60); no difference in advanced adenomas (0.03 per colonoscopy in both groups)	GRADE used; evidence rated high for ADR/PDR and advanced adenomas; four of five trials had at least one high-risk-of-bias domain	False positives in 4 trials: 7.1–20.1% (mean 11.2%); withdrawal about 30 s longer with AI	Geographic restriction; mostly unblinded/single-blind trials; per-protocol analyses; limited cancer data	Strong evidence for smaller/nonadvanced lesions, but no demonstrated gain in advanced adenoma detection in this early evidence base.
**Lou et al. (2023)** [93]	Systematic review and meta-analysis of 33 RCTs; Asia and Western settings	27,404 randomized participants	Multiple CADe and CAQ systems; commercial and investigational	Standard colonoscopy	AMR, ADR, APC, PMR, PDR, PPC, procedure time, adverse events	AMR RR 0.495 (0.390–0.627); ADR RR 1.242 (1.159–1.332); APC IRR 1.390 (1.277–1.513); PDR RR 1.238 (1.158–1.323); inspection time + 0.33 min	RoB 2, GRADE, prediction intervals, meta-regression and sensitivity analyses; evidence quality very low to moderate	Pooled false-positive proportion 12.2% (95% CI 7.5–16.9%)	High heterogeneity; prediction intervals crossed no effect for several outcomes; publication bias for ADR/PDR/APC; aggregate-level subgroup analyses	Average detection benefit is supported, but effect size varies materially by setting, baseline performance and study design; long-term cancer outcomes remain unproven.
**Xu et al. (2021)** [94]	Prospective multicenter parallel RCT; 6 Chinese centers	2352 analyzed (1177 AI; 1175 control)	Investigational RetinaNet/FPN/ResNet-50 real-time detector	Conventional white-light colonoscopy	PDR; PPP, PPC, PPC-Plus; lesion characteristics	PDR 38.8% vs. 36.2% (*p* = 0.183); PPC-Plus 0.5 vs. 0.4 (*p* = 0.024); more diminutive polyps (76.0% vs. 68.8%) and flat polyps (5.9% vs. 3.3%)	Prospective multicenter randomization; system tested on 117,048 frames; sensitivity 81.9%, specificity 98.4%	No system-related adverse events; FP burden not fully quantified in clinical trial	Per-protocol analysis; ADR not assessed for all patients; effects differed by center; partial histology only	No significant improvement in the primary endpoint; benefit was concentrated in additional, diminutive and flat polyps.
**Xu et al. (2023)** [95]	Prospective multicenter single-blind parallel RCT; 6 screening centers	3059 asymptomatic participants (1519 AI; 1540 control)	Eagle-Eye real-time CADe on same monitor	Conventional colonoscopy	Overall ADR; APC; advanced ADR; endoscopist-experience subgroups; withdrawal time	ADR 39.9% vs. 32.4% (*p* < 0.001); advanced ADR 6.6% vs. 4.9% (*p* = 0.041); APC 0.59 vs. 0.45; withdrawal 8.25 vs. 7.78 min	Large multicenter RCT; ITT and per-protocol analyses; pathology blinded; expert and nonexpert strata	Immediate adverse events: none; FP rate not reported	Single-blind; longer withdrawal with AI; no improvement in CRC or SSL detection; surrogate endpoints	Convincing screening-population ADR gain, including advanced adenomas, but the absolute advanced-lesion gain was small and no patient-important outcome was measured.
**Wang et al. (2020)** [96]	Single-center double-blind randomized sham-controlled trial; China	962 analyzed (484 CADe; 478 sham)	EndoScreener deep-learning CADe with TensorRT	Sham alerts on non-polyp structures	ADR; PDR; adenomas/polyps per colonoscopy	ADR 34.1% vs. 27.6%, OR 1.36 (1.03–1.79); PDR 52% vs. 37%, OR 1.86; APC 0.58 vs. 0.38; PPC 1.04 vs. 0.64	Double-blind sham design minimized operational bias; independent post-trial review of missed lesions	48 consistent false detections (about 0.1/colonoscopy); no complications	Single center; senior endoscopists; most incremental lesions were diminutive; effect on interval CRC unknown	Methodologically strong short-term efficacy evidence, mainly for small lesions; clinical significance beyond surrogate detection remains uncertain.
**Glissen Brown et al. (2022)** [84]	US multicenter single-blind randomized tandem colonoscopy trial	223 analyzed (113 CADe-first; 110 HDWL-first)	EndoScreener, SegNet-based deep-learning CADe	High-definition white-light colonoscopy in opposite tandem sequence	AMR; SSL miss rate; first-pass APC and ADR	AMR 20.12% vs. 31.25% (*p* = 0.0247); SSL miss 7.14% vs. 42.11% (*p* = 0.0482); first-pass APC 1.19 vs. 0.90 (*p* = 0.0323); ADR difference not significant	Four academic centers; randomized tandem design; pathologists blinded	203 FP alerts across CADe passes; 3 false negatives among 315 CADe-procedure polyps	Small sample; tandem/order and observer effects; high-performing academic endoscopists; not powered for ADR	Supports reduced miss rates, including SSLs, but precision is limited for rare outcomes and tandem findings may not fully generalize to routine practice.
**Mangas-Sanjuan et al. (2023)** [97]	Multicenter parallel RCT in Spanish FIT-positive screening program	3213 analyzed (1610 CADe; 1603 control)	GI Genius v2.0	Colonoscopy without CADe	Advanced colorectal neoplasia detection; advanced lesions per colonoscopy; ADR and lesion subgroups	Advanced neoplasia 34.8% vs. 34.6%, aRR 1.01; ADR 64.2% vs. 62.0%, aRR 1.06; smaller, nonpolypoid and proximal lesions per colonoscopy	Adequately powered for advanced neoplasia; 6 centers; adjusted mixed models; pathology blinded	FP/FN data not collected; withdrawal longer with CADe	Very high baseline ADR and FIT-positive population; endoscopists unblinded; secondary analyses used stringent multiplicity threshold	High-quality negative trial for advanced neoplasia. CADe increased lesion counts modestly but did not improve the main clinically relevant detection endpoint.
**Park et al. (2024)** [98]	Prospective multicenter randomized trial; 6 Korean tertiary centers	805 analyzed (437 AI; 368 control)	RetinaNet/ResNet-50; still-image plus video transfer learning; 5-frame post-processing	Colonoscopy without AI	ADR and PDR; model sensitivity and FP/frame	PDR 62% vs. 52% (*p* = 0.01); ADR 35% vs. 28% (*p* = 0.03); model sensitivity 97.87%; FP/frame reduced from 0.0647 to 0.0078 after training/post-processing	10-fold cross-validation for still images; separate video training and 8-video evaluation; randomized clinical comparison	Post-processing substantially reduced FP/frame; withdrawal time not significantly different	Unequal group sizes after exclusions; baseline BMI imbalance; clinical analysis and regression reporting are unconventional; tertiary setting	Shows both technical FP reduction and a modest clinical detection gain, but reporting limitations reduce certainty about effect size.
**Lau et al. (2024)** [77]	Single-center single-blind parallel RCT; Hong Kong; trainees only	766 analyzed (386 CADe; 380 control); 22 trainees <500 procedures	ENDO-AID OIP-1 (Olympus)	Standard high-definition colonoscopy	ADR; lesion-size/location ADR; APC; non-neoplastic resection; workflow	ADR 57.5% vs. 44.5%, adjusted RR 1.41; APC 1.48 vs. 0.86; no advanced ADR benefit; non-neoplastic resection 52.1% vs. 35.0%	Stratified randomization by age, sex and trainee experience; outcome assessors blinded; covariate-adjusted analysis	FP signal rate 23.8%; mean 1.1 FP/colonoscopy; withdrawal + 1.2 min	Single center; operators unblinded; high baseline ADR; supervisor presence; missed/FP events operator-reported	Clear trainee detection benefit, especially for small/nonpedunculated lesions, offset by more non-neoplastic resections and longer withdrawal.
**Chung et al. (2024)** [99]	Prospective nonrandomized comparison in routine Korean practice	3047 subjects: control 1591; high-FP system 763; low-FP system 693	Two YOLOv4 CADe systems; A high FP (3.2%), B low FP (0.6%)	Standard colonoscopy and head-to-head system comparison	ADR, APC, non-true lesion resection rate (NTLR)	ADR 44.3% control, 43.4% A, 50.4% B; adjusted RR B vs. control 1.11; APC 0.75, 0.83, 0.90; NTLR 23.8%, 29.2%, 21.3%	Same test set and endoscopists; both systems 100% per-lesion sensitivity; multivariable adjustment	A: 3.28 FP/min; B: 1.24 FP/min; longer 0.1–1 s FP alerts concentrated in A	Nonrandomized room-based allocation; no endoscopist blinding; different training composition/post-processing; residual confounding	Suggests that alarm burden can negate clinical benefit and increase unnecessary resection, but causality is limited by the nonrandomized design.
**Jeon et al. (2026)** [86]	Prospective multicenter single-blind randomized trial; 2 Korean tertiary centers	998 analyzed (497 CADe; 501 control)	ENAD-DET-01; Kalman-filtered real-time CADe	Conventional colonoscopy	ADR; PDR; APC; predictors of adenoma detection	ADR 52.3% vs. 36.1%; PDR 72.2% vs. 54.5%; APC 1.2 vs. 0.8; adjusted OR for CADe 1.914	Center-stratified concealed block randomization; trained on 245,978 images from 18,213 polyps; per-lesion sensitivity 100%, FP-frame rate 0.6%	Clinical false-positive occurrence reported as 8.7%; withdrawal 9.3 vs. 8.5 min	Endoscopists unblinded; withdrawal time differed; miss rate not measured; no significant SSL or cancer benefit	Large short-term ADR/PDR benefit after adjustment, but longer inspection and absence of miss-rate or hard-outcome data limit causal and clinical interpretation.
**Rex et al. (2022)** [73]	Prospective single-endoscopist observational evaluation; US high-detector setting	289 colonoscopies; 1040 resected lesions	First FDA-approved CADe device (commercial system)	High-detecting endoscopist performance with and without AI-first lesions	Incremental ADR/APC; lesion-level detection timing; missed lesion phenotype	ADR in patients ≥ 50 increased 52.9% to 60.6% when AI-first lesions included; 21/1040 lesions not detected by AI; missed lesions larger and flatter	Prospective lesion-level categorization; detailed phenotype of CADe misses	Not a randomized comparison; FP burden not the primary outcome	Single expert, subjective timing categories, surveillance-heavy case mix, potential conflict of interest; selection/generalizability concerns	Demonstrates incremental value even at high baseline ADR while exposing an important weakness for larger flat lesions; hypothesis-generating rather than comparative efficacy evidence.
**Brand et al. (2022)** [72]	Retrospective frame-by-frame analysis of full-length colonoscopy videos; 2 German tertiary centers	111 videos; 1,793,371 frames; 118 polyps	GI Genius commercial CADe (March 2020 software)	Expert frame annotation of raw and CADe video pairs	FP number/duration; per-polyp and per-frame sensitivity; time to first detection	100% per-polyp sensitivity; per-frame sensitivity 47.73%; 101 ± 88 FP activations/colonoscopy; mean first detection 1.69 s; 6-frame threshold reduced FPs 85.54%	Full-length real-world videos; detailed frame-level ground truth and threshold analysis	Most FPs lasted 1–2 frames; thresholds > 3 frames began to miss some polyps, especially flat lesions	Retrospective; selected adequate-prep cases; histology unavailable; one expert annotator; hyperplastic rectosigmoid polyps excluded	Excellent lesion-level sensitivity can coexist with unstable frame-level detection and heavy transient alarm burden; thresholding requires a sensitivity trade-off.
**Krenzer et al. (2023)** [100]	Technical development and external benchmark validation; clinical-ready prototype	Training: 506,338 images; independent EndoData test plus CVC-VideoClinicDB benchmark	Open-source ENDOMIND-Advanced; YOLOv5 + real-time REPP temporal post-processing	Public video benchmark and independent multicenter-derived test data	Precision, recall, F1, mAP, speed; prototype integration	F1 90.24% on CVC-VideoClinicDB; selected YOLOv5 model achieved F1 88.70%, mAP 86.44%, 43 FPS in cross-validation	Public benchmark, independent clinic test set, 5-fold CV, cross-center and multi-vendor training data	Temporal post-processing designed to suppress unlinked FP boxes; no patient-level ADR/PDR trial	No prospective comparative clinical outcomes; benchmark annotations and dataset composition may not represent all routine settings	Strong technical and reproducibility contribution, but clinical benefit, safety and workflow impact remain unvalidated in a controlled patient trial.
**Repici et al. (2020)** [101]	Prospective multicenter randomized parallel trial; Italy	685 randomized participants (341 CADe; 344 control)	GI Genius real-time CADe	High-definition white-light colonoscopy	ADR and APC; lesion-size and morphology subgroups	Higher ADR and APC with CADe, driven mainly by additional small and diminutive adenomas; no long-term colorectal cancer outcome assessment	Multicenter randomized design; histopathology reference	False-positive burden was not a primary clinical endpoint in this trial	Endoscopists unblinded; surrogate detection endpoints; long-term CRC benefit not assessed	Supports clinical efficacy in a Western multicenter setting, while leaving uncertain whether extra small-lesion detection improves patient-important outcomes.

**Table 7:** Clinical and technical evidence for CADx and autonomous systems in AI-assisted colonoscopy.

Study (Year)	Evidence Type/ Setting	Population/ Sample	CADx System/ Model	Imaging/ Task	Training Dataset Characteristics	Comparator/Reference	Key Quantitative Findings	Independent External Validation	Regulatory Scope (FDA/CE)	Workflow/Confidence	Main Limitations	Critical Analysis and Synthesis
**Byrne et al. (2019)** [102]	Technical development/validation; unaltered colonoscopy videos	Diminutive colorectal polyps evaluated from colonoscopy video frames	Deep-learning CADx	Real-time adenoma vs. hyperplastic classification from standard video	60,089 NBI frames reported for model development in the evidence synthesis; dataset composition and center diversity incompletely reported	Histopathology; expert optical diagnosis	High diagnostic performance reported for classifiable diminutive polyps; performance depends on system providing a diagnosis	No independent prospective live multicenter validation in this report	NR; no clearance claim made in the paper	Real-time output; non-diagnostic/low-confidence cases affect applicability	Selected videos; spectrum and selection bias; limited live-clinical evidence	Proof of technical feasibility, but not sufficient alone for diagnose-and-leave or resect-and-discard implementation.
**Bang et al. (2021)** [103]	Systematic review/meta-analysis of CADx diagnostic accuracy	Multiple studies of diminutive colorectal polyps; heterogeneous per-image/per-polyp samples	Mixed CADx platforms and algorithms	Optical characterization, mainly neoplastic vs. non-neoplastic	Not applicable; underlying studies used heterogeneous, often curated training datasets	Histopathology	High pooled AUC/sensitivity/specificity reported, but estimates combine different platforms and study units	Variable; many included studies were internal or selected-dataset validations	Not applicable; review does not establish product-specific clearance	Reporting of confidence thresholds and non-diagnostic cases varied	Substantial heterogeneity, publication bias risk, selected images and overlapping evidence	Supports promise of CADx, but pooled performance should not be treated as proof that any single platform is ready for autonomous management.
**Song et al. (2020)** [104]	Single-center development with retrospective test set and prospectively acquired real-time still-image test	1169 polyps total; training 624, test I 182, test II 363 polyps	DenseNet-201 CAD (after comparison with ResNet-50)	Near-focus NBI; SP vs. BA/MSMC vs. DSMC	624 images expanded to 12,480 patches; expert-acquired images; oversampling and augmentation; fivefold cross-validation	Histopathology; 3 experts and 3 trainees	Accuracy 81.3% and 82.4% in test sets; CAD better than trainees and comparable/slightly inferior to experts; DSMC sensitivity 58.8% and 62.1%; inference 0.02–0.04 s	Prospective second test set, but same center and image-acquisition ecosystem; no independent-center validation	NR; investigational system	Real-time transfer of 1–5 still images via PACS; trainee performance improved with CAD assistance	Single center; still images; all images acquired by experts; few DSMC cases; no external-center test	Clinically relevant treatment-oriented classes and prospective workflow test are strengths; weak DSMC sensitivity and lack of external validation limit autonomous treatment planning.
**Zhou et al. (2020)** [92]	Retrospective multicohort diagnostic study; one internal and two external test hospitals	Training: 12,179 patients/464,105 images; tests: 2263 patients/84,615 images	CRCNet, DenseNet-169 with focal loss and Grad-CAM	White-light images; patient-level CRC vs. non-CRC	Single-center training; large dataset; 3176 CRC and 9003 controls; extensive augmentation; 56.1% of training controls had pathology	Pathology; five skilled endoscopists	Patient-level AUPRC 0.882, 0.874, 0.867; accuracy 87.3%, 91.6%, 98.0% across test sets; image-level AUPRC 0.990–0.997	Yes: two external hospitals, but all cohorts were retrospective and from northern China	NR; investigational model	Patient score aggregated across multiple images; Grad-CAM explanation	Static images; training from one center; regional population; binary malignancy task; rare malignancies excluded; no prospective outcome study	Large dataset and hospital-level external testing strengthen discrimination evidence, but this is CRC classification rather than routine diminutive-polyp CADx and requires prospective validation.
**Houwen et al. (2023)** [105]	Prospective multicenter real-time comparative study	194 FIT-positive patients; 423 diminutive polyps; 20 screening endoscopists from 8 hospitals	POLAR	Non-magnified NBI; adenoma/SSL/HPP and neoplastic vs. non-neoplastic	2637 annotated images from 1339 polyps collected at 8 hospitals; MS-COCO pretraining; YOLOv4 localization plus quality checks and gridSIFT classifier	Histopathology; blinded screening endoscopists	Accuracy 79.4% vs. 83.0% for endoscopists (*p* = 0.10); sensitivity 89.4%, specificity 37.8%; success 98.3%; SSL sensitivity 17.1%; mean 1.4 s/image	Clinical validation across multiple hospitals, but training and validation were within the same project/network and NBI platform	NR; investigational POLAR system	Maximum 3 images; confidence/quality feedback; prediction hidden from endoscopists	Low specificity and rectosigmoid NPV; poor SSL sensitivity; NBI-only, image-based; no central pathology review; clustering not included in sample-size calculation	Methodologically strong real-time multicenter benchmarking, but performance did not meet standards needed for safe stand-alone optical diagnosis.
**Djinbachian et al. (2024)** [106]	Randomized controlled trial	Prospective colorectal-polyp optical diagnosis population	Autonomous AI vs. AI-assisted human optical diagnosis	Real-time optical diagnosis and surveillance assignment	Product-specific training dataset details were not fully transparent in the summarized evidence	Histopathology; pathology-concordant surveillance intervals	Autonomous AI reported non-inferior to AI-assisted human diagnosis, while overall optical accuracy remained limited	Prospective clinical trial; independent external generalizability beyond participating centers remains uncertain	Regulatory clearance was not specified in the evaluated source	Autonomous output compared with human-AI workflow	Non-inferiority does not establish adequate absolute safety; management errors remain clinically important	Important direct test of autonomy, but non-inferiority to a modest-performing comparator is not equivalent to meeting PIVI/SODA thresholds.
**Hassan et al. (2023)** [89]	Head-to-head comparative research letter of commercial optical-diagnosis systems	Common colorectal-polyp dataset evaluated across multiple commercial systems	Multiple commercial CADx systems	Leaving-in situ optical diagnosis of diminutive rectosigmoid polyps	Each product used different proprietary training/validation datasets; clinical composition largely undisclosed	Histopathology; guideline-based surveillance intervals	System performance and agreement differed; availability of a commercial product did not guarantee uniform threshold attainment	Head-to-head testing on a common dataset improves comparability, but it is not a prospective live multicenter deployment study	Commercially available systems discussed; regulatory indications vary by product and jurisdiction	Offline/common-dataset comparison; no routine workflow assessment	Research-letter format; sample and dataset constraints; proprietary training data; expert-context effects	Valuable comparative evidence showing that CADx systems are not interchangeable; regulatory and clinical claims require platform-specific documentation.
**Vadhwana et al. (2023)** [107]	Systematic review and meta-analysis of prospective real-time histology studies	13 studies; 2958 patients and 5908 lesions; 6 studies in direct human-vs-CAD comparison	Six heterogeneous AI technology families	Real-time histological prediction using NBI, WLI, BLI, AFI, ESS and related systems	Not applicable; underlying studies had widely varying training sets, several incompletely reported	Histopathology; endoscopist diagnosis	Across direct comparisons, endoscopist sensitivity favored CAD but difference was not significant (*p* = 0.43); platform-specific performance varied widely	Included prospective studies, but cross-platform external validation was inconsistent	Not applicable; review does not verify product-specific FDA/CE indications	Real-time focus is a strength; workflows differed substantially	Clinical and technical heterogeneity; pooled bivariate/HSROC analysis not feasible; only 13 studies; platform iterations differed	Best interpreted as an evidence map: prospective CADx is promising, but current systems were not consistently superior to experts and are not one uniform intervention.
**Nazarian et al. (2021)** [108]	Systematic review/meta-analysis of detection and characterization evidence	48 studies overall; characterization analyses included up to 4001 patients depending on unit of analysis	Mixed CADe and CADx systems	Detection and histological characterization across still images, clips and real-time trials	Not applicable; underlying datasets varied greatly in size, modality and quality	Histopathology/diagnostic standard; standard colonoscopy for RCT detection outcomes	Characterization: pooled sensitivity 0.94, specificity 0.87, accuracy 0.91 across 20 studies; detection RCTs increased PDR (OR 1.75) and ADR (OR 1.53)	Variable; mostly preclinical/retrospective characterization evidence	Not applicable; review cannot establish product-specific clearance	Real-time characterization evidence was limited; many studies excluded poor-quality images	Very high heterogeneity; combines CADe and CADx; mixed per-image/per-polyp/per-patient units; overlap and spectrum bias	Useful broad benchmark, but the characterization estimates cannot be transferred directly to a specific CADx platform or autonomous clinical strategy.
**Hann et al. (2021)** [109]	Narrative review of status and limitations	No primary patient sample	CADe/CADx review	Detection and characterization in colonoscopy	Not applicable	Published technical and clinical literature	Concludes CADe evidence was more mature and CADx required further development and validation	Not applicable	Review predates later product versions; regulatory status should be interpreted at the product and jurisdiction level	Discusses workflow, false positives, generalizability and implementation	Narrative selection; no formal pooled analysis or reproducible appraisal; rapidly dated field	Useful contextual critique, but should not be weighted as primary validation evidence and should be separated from trials and meta-analyses.

Table 6 and Table 7 synthesize current evidence on CADe, CADx, and autonomous AI in colonoscopy. CADe is the most clinically mature application and generally improves surrogate detection outcomes such as ADR, PDR, and AMR, although benefits mainly involve small or diminutive lesions and effects on advanced neoplasia, sessile serrated lesions, CRC incidence, and mortality remain uncertain. False positives, longer withdrawal time, and non-neoplastic resections may limit clinical value. CADx and autonomous systems show promising accuracy for optical diagnosis and surveillance decisions, but evidence is more heterogeneous and less mature. Differences in datasets, imaging methods, thresholds, validation, regulation, and real-time testing limit generalizability. Overall, AI-assisted colonoscopy should be implemented cautiously, with CADe better supported than CADx or autonomous systems for independent decision-making; further prospective multicenter validation and patient-centered outcome data are needed.

## 7. Model Architectures

Most polyp detection models are based on object detection algorithms, which define the extent of the lesion using bounding boxes. Faster R-CNN architectures are highly accurate but computationally intensive, while one-stage models (SSD, RetinaNet, YOLO) allow for quick evaluation and can achieve real-time detection. Transformer-based models have even better detection rates, particularly for small polyps and are faster [92,102,110,111]. Segmentation models: U-Net, DeepLab, Mask R-CNN, PraNet and transformer architectures have been employed for the accurate delineation of polyps. Segmentation is more informative than detection, as it enables an estimate of lesion size and resectability; however, the accuracy tends to be poorer in the case of flat, difficult-to-see, or partially occluded lesions.

Characterization models (CADx): deep neural networks as learning approaches, including convolutional neural networks and transformer-based models, can characterize colorectal lesions based on their neoplastic features and invasion depth using various endoscopic imaging modalities. Their potential clinical applications include optical diagnosis, resect-and-discard strategies, and assessment of lesions requiring advanced endoscopic (endoscopic submucosal dissection (ESD)) or surgical management [108,112].

## 8. Datasets, Annotation, and External Validation

Despite the rapid growth of research on AI-assisted colonoscopy, the current literature and evidence remain limited by methodological heterogeneity, which complicates comparisons across studies and limits the generalizability of findings [105]. Various study designs, including parallel and tandem approaches, have been used to evaluate AI-assisted colonoscopy [86,92]. Differences in outcome measures, image datasets, image quality, endoscopic equipment, bowel preparation, and clinical settings can contribute to variability between studies and limit direct comparisons [77,85]. Differences in image datasets, ranging from large databases containing hundreds of thousands of images to single-patient case studies, as well as variations in image quality and imaging modality, may also affect AI performance [96,106]. Additionally, variations in bowel preparation, endoscopy systems, and camera technology may affect study outcome measures and influence AI performance [95,102]. Machine-learning models may be prone to overfitting, particularly when trained on limited or highly selected datasets, which can result in reduced generalizability to new images and real-world clinical settings [113].

Additional work, especially prospective multicenter trials, comparative effectiveness trials, and external validations, is necessary to improve beyond current shortcomings [108].

## 9. Clinical Implications, Limitations and Risks of AI-Assisted Colonoscopy

A major limitation of the current evidence is that most studies focus on intermediate outcomes, such as adenoma detection and miss rates, while the long-term impact of AI on colorectal cancer incidence and mortality remains uncertain. Some authors, such as Wang, state that the impact of CADe on interval CRC needs to be further examined, while Lou emphasizes the need to assess the long-term benefit in reducing the incidence of CRC [84,93,96]. Even where an increase in ADRs is assumed to lead to better prevention, a direct causal link between AI-assisted detection and a reduction in mortality has not yet been confirmed [91,114]. Increased detection of predominantly diminutive polyps may lead to overdiagnosis and more frequent surveillance colonoscopies, increasing the burden on patients and healthcare resources [114]. Future research should focus on further improving CADe systems and determining whether these improvements translate into meaningful reductions in the burden of colorectal cancer.

Similar concerns are expressed by reviews that emphasize the possibility of operator deskilling, the need for additional algorithmic improvement and the still unclear long-term effect on patients [84,103]. That is why future studies should not be satisfied only with proving that AI “discovers more”, but also with the question of what happens to patients after it discovers more [84,114]. The possibility of multimodality is particularly interesting, because reviews indicate that AI can be associated with magnifying narrow-band imaging, endocytoscopy, confocal endomicroscopy (CE) and other techniques to improve the characterization of lesions [72,104]. One of the risks of AI-Assisted colonoscopy is false classifications, which means that it may occasionally fail to detect or correctly characterize clinically relevant lesions, potentially leading to missed pathology or inappropriate management decisions. Prolonged exposure to AI-assisted colonoscopy may alter endoscopist behavior, potentially impairing independent adenoma detection when AI is no longer available, raising concerns about a potential deskilling effect [115].

The development and role of AI models that integrate morphological, histological, and clinical features could enable more accurate patient risk stratification and support individualized surveillance intervals, potentially reducing unnecessary follow-up procedures and healthcare burden [77,78]. However, this will require further improvements in the calibration and interpretability of AI models, as well as confirmation of their reliability through external validation studies that include different populations and polyp prevalence [86,88]. Otherwise, multimodality may increase system complexity without providing a corresponding clinical benefit [108,116].

## 10. Future Perspectives

**Figure 4:**
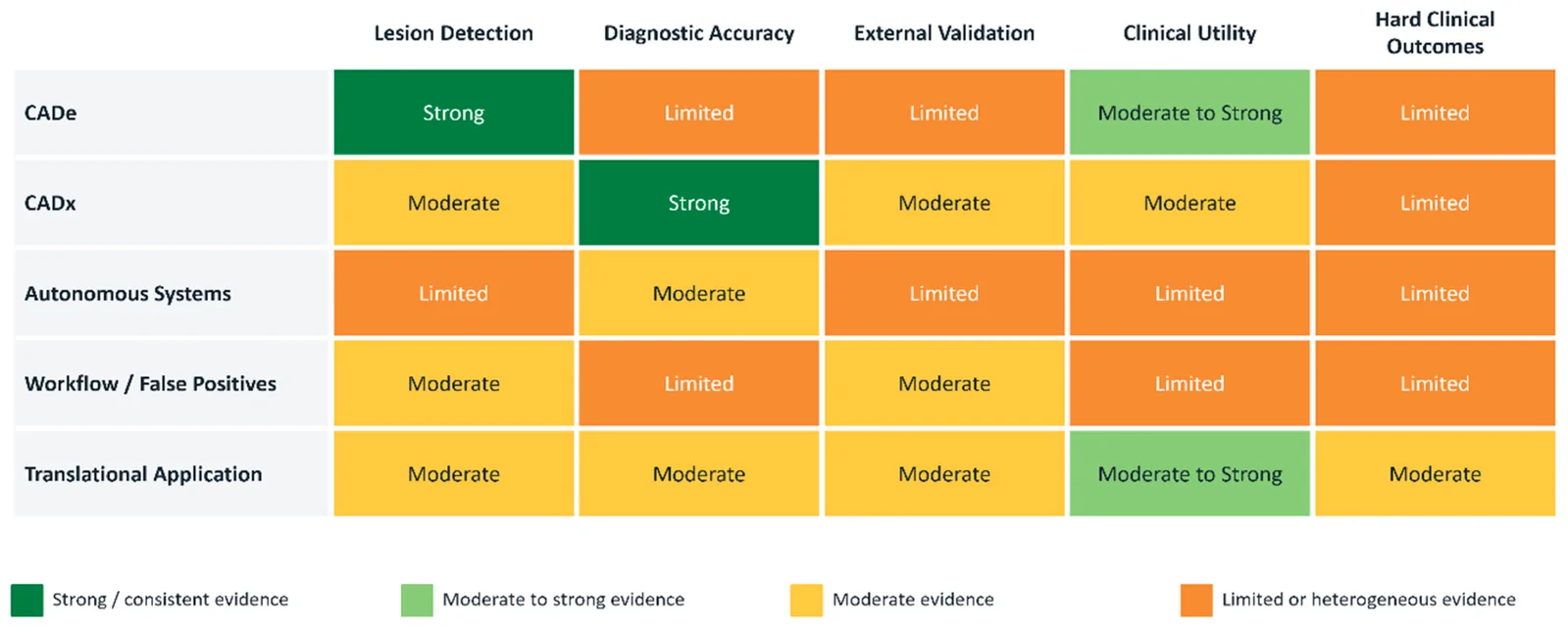
Descriptive evidence map.

There has been a shift from largely experimental to realistic application of colonoscopy AI, but the extent of whole system standardization and use is not fully resolved (14). Among the different AI applications in colonoscopy, CADe currently has a relatively well-established evidence base, whereas CADx and autonomous systems continue to face methodological, practical, and regulatory challenges [78,84,93]. Figure 4 is presented as a descriptive heatmap that summarizes the available literature across different AI applications in colonoscopy, without implying a formal assessment of the strength or quality of the evidence (Figure 4).

CADe has a relatively strong clinical evidence base for improving ADR, while CADx has mainly been studied for optical diagnosis and lesion characterization. However, evidence for patient-important outcomes, including CRC incidence, mortality, and post-colonoscopy colorectal cancer (PCCRC), remains limited, as long-term prospective data are still lacking [74,93,96,112,117,118,119,120,121].

Future research should focus on developing more advanced AI-assisted systems aimed at reducing the number of clinically significant missed lesions and, ultimately, the risk of CRC following colonoscopy. Development should gradually move beyond improving detection toward CRC prevention, demonstrating the potential of AI, supported by pragmatic clinical trials, prospective registries, and health-outcomes research.

## 11. Conclusions

Overall, this scoping review shows that CADe technologies currently offer the highest level of evidence among AI systems in colonoscopy, while CADx, segmentation, AI-assisted lesion detection, and AI-assisted clinical decision-making and educational tools are still at different levels of maturity. Many reports describe increased adenoma detection and reduced lesion miss rates for CADe; however, the effect on clinical outcome and future CRC incidence is yet to be determined. Therefore, future studies should investigate the impact of AI on cancer prevention in combination with analysis of safety, cost-effectiveness, implementation strategies and health benefits. In particular, future research requires a careful approach to prospective, registry-based, and health technology pricing for meaningful progress in this area.

## Figures and Tables

**Table 1:** Inclusion and exclusion criteria.

Inclusion Criteria	Exclusion Criteria
Adult patients aged ≥18 years undergoing colonoscopy	Animal or in vitro studies
Studies evaluating artificial intelligence applications in colonoscopy, including CADe, CADx, CAQ, segmentation, and decision-support systems	Pediatric populations
Studies addressing colorectal polyps, adenomas, precancerous lesions, or colorectal cancer detection and characterization	Non-English-language publications
Studies reporting clinical, technical, or implementation-related outcomes, including PDR, ADR, AMR, PMR, optical diagnosis performance, quality indicators, validation metrics, or implementation outcomes	Studies focused exclusively on non-colorectal malignancies, non-endoscopic applications, or non-AI endoscopic technologies
Studies comparing AI-assisted colonoscopy with routine colonoscopy	Protocols without results, duplicate reports, or publications for which essential methodological or outcome information could not be obtained or verified
Randomized controlled trials, observational studies, comparative studies, systematic reviews, meta-analyses, clinical guidelines, consensus statements, and technical validation studies	Editorials, letters, commentaries, conference abstracts, and other non-original publications lacking sufficient methodological or outcome data

**Table 2:** Search strategy and number of retrieved records from January 2010 to June 2026.

Database	Search String	Number of Results
PubMed	((“Colorectal Neoplasms”[Mesh] OR “Colonic Polyps”[Mesh] OR “Intestinal Polyps”[Mesh] OR “colorectal polyp*”[tiab] OR “colorectal cancer*”[tiab] OR “colorectal neoplasm*”[tiab] OR “colorectal adenoma*”[tiab] OR “colon polyp*”[tiab] OR “colonic polyp*”[tiab] OR “colon adenoma*”[tiab] OR “colonic adenoma*”[tiab]) AND (“Artificial Intelligence”[Mesh] OR “Machine Learning”[Mesh] OR “Deep Learning”[Mesh] OR “Pattern Recognition, Automated”[Mesh] OR “artificial intelligence”[tiab] OR “machine learning”[tiab] OR “deep learning”[tiab] OR “computer-aided diagnos*”[tiab] OR “computer-assisted diagnos*”[tiab] OR “computer-aided detect*”[tiab] OR “computer-assisted detect*”[tiab] OR “CADe”[tiab] OR “CADx”[tiab] OR “polyp segmentation”[tiab] OR “convolutional neural network*”[tiab] OR “CNN”[tiab]) AND (“Colonoscopy”[Mesh] OR “Endoscopy, Gastrointestinal”[Mesh] OR “colonoscop*”[tiab] OR “endoscop*”[tiab]) AND (“Quality Indicators, Health Care”[Mesh] OR “clinical integration”[tiab] OR “adenoma detection rate*”[tiab] OR “adenoma detection”[tiab] OR “adenoma miss rate*”[tiab] OR “polyp detection rate*”[tiab] OR “polyp miss rate*”[tiab] OR “quality indicator*”[tiab] OR “quality metric*”[tiab] OR “withdrawal time”[tiab]))	393
Google Scholar	intitle:(colonoscopy OR polyp OR adenoma) AND (CADe OR CADx OR “computer-aided detection” OR “artificial intelligence”) AND (“adenoma detection rate” OR ADR OR “polyp detection rate” OR PDR)	4830
Crossref	(“colorectal polyp” OR “colorectal cancer” OR “colorectal neoplasm” OR “colon polyp” OR “adenoma”) AND (“artificial intelligence” OR “AI” OR “machine learning” OR “deep learning” OR “computer-aided detection” OR “computer-aided diagnosis” OR “CADe” OR “CADx” OR “polyp segmentation”) AND (“colonoscopy” OR “endoscopy”) AND (“clinical integration” OR “adenoma detection rate” OR “ADR” OR “adenoma miss rate” OR “AMR” OR “quality indicators”)	11,512

**Table 3:** Key quality benchmark indicators for colonoscopy.

Key Performance Indicators	Benchmark	Definition
**Cecal Intubation Rate (CIR)**	**≥95%**	Proportion of colonoscopies in which the cecum is successfully intubated.
**Adequate Bowel Preparation Rate**	**≥85%**	Proportion of colonoscopies with bowel preparation adequate for detecting polyps >5 mm.
**Adenoma Detection Rate (ADR)**	**≥35%** **overall** **≥40%** **men** **≥30% women**	Proportion of screening colonoscopies in which at least one adenoma is detected.
**Polyp Detection Rate (PDR)**	**≥30%**	Proportion of colonoscopies in which at least one polyp of any histology is detected.
**Serrated Lesion Detection Rate (SLDR)**	**≥10%**	Proportion of screening colonoscopies in which at least one serrated lesion is detected.
**Withdrawal Time**	**≥6 min**	Mean withdrawal time from the cecum to the rectum during normal colonoscopy.
**Complication Rate**	**≤0.3%**	Rate of major complications (bleeding, perforation, cardiopulmonary events) within 30 days after colonoscopy.
**Post-Polypectomy Bleeding Rate**	**≤1%**	Rate of clinically significant bleeding requiring endoscopic, radiologic, or surgical intervention.
**Perforation Rate**	**≤0.1%**	Rate of perforations requiring endoscopic, surgical, or hospital management.
**Appropriate Surveillance Rate**	**≥90%**	Proportion of patients assigned surveillance intervals consistent with current clinical guidelines.

Benchmark sources: American Society for Gastrointestinal Endoscopy (ASGE), American College of Gastroenterology (ACG), European Society of Gastrointestinal Endoscopy (ESGE), and British Society of Gastroenterology (BSG).

**Table 4:** Functional taxonomy of AI systems used in colonoscopy.

AI Function	Clinical Task	Typical Output	Key Validation Endpoints
CAQ (Computer-Aided Quality Assessment)	Quality assessment of colonoscopy	Withdrawal speed monitoring, blind-spot alerts, bowel preparation assessment, mucosal exposure score	Improvement in examination quality, completeness of mucosal inspection, adherence to quality indicators
CADe (Computer-Aided Detection)	Detection of colorectal lesions	Real-time visual highlighting, bounding box, audible or visual alerts	ADR, PDR, adenomas per colonoscopy (APC), AMR, false-positive activation rate
CADx (Computer-Aided Diagnosis)	Characterization of detected lesions	Histological prediction, confidence score, neoplastic risk classification, optical diagnosis	Sensitivity, specificity, positive predictive value (PPV), negative predictive value (NPV), high-confidence optical diagnosis rate
Segmentation	Lesion boundary delineation	Pixel-level lesion mask, lesion contour, size estimation	Dice similarity coefficient (DSC), Intersection over Union (IoU), boundary accuracy, lesion size estimation accuracy
Decision Support	Clinical management guidance	Recommendations for endoscopic resection, surveillance interval, ESD, surgery, or follow-up	Guideline adherence, patient safety, clinical outcomes, workflow efficiency, cost-effectiveness

**Table 5:** Commercial and near-commercial AI platforms used in colonoscopy.

Platform	Developer/ Ecosystem	Training Dataset Characteristics	Independent External Validation	FDA/CE Status and Exact Scope	Main Indication	Critical Appraisal
**GI Genius** [72,73,74,75]	**Cosmo Intelligent Medical Devices; distributed by Medtronic**	The commercial training set is incompletely disclosed. Available publications indicate a lesion-rich video/image library dominated by diminutive lesions; patient numbers, center mix, demographic diversity, and representation of serrated or large flat lesions are not consistently reported.	Independent evaluations in routine practice reported higher adenomas per procedure and ADR. Expert-operator and frame-by-frame analyses also showed detection gains, but identified missed large flat lesions and brief false-positive activations.	FDA De Novo authorization and subsequent 510 (k) clearances identify GI Genius as a gastrointestinal lesion software detection system for real-time detection of colonic mucosal lesions during standard white-light colonoscopy. It is not intended to characterize lesions or replace clinical decision-making. CE-marked CADe use is established in Europe; CADx or autonomous histological diagnosis should not be assumed unless specifically documented.	Real-time CADe during colonoscope insertion and/or withdrawal as an adjunct to, not a replacement for, endoscopist judgment.	This platform has the broadest clinical evidence among the reviewed systems, including pragmatic data. Benefits are driven mainly by additional small lesions, while false-positive burden, performance for large or flat lesions, and training-data transparency remain important limitations.
**CAD EYE** [58,76]	**Fujifilm**	Exact proprietary development dataset is not fully disclosed in the reviewed clinical papers. The system is integrated with Fujifilm imaging and supports WLI/LCI detection and BLI characterization. Available studies include small colorectal lesions, neoplastic and hyperplastic classes, and magnified or non-magnified BLI, but do not provide a complete patient-level training-data profile.	Independent prospective and retrospective evaluations show high detection and characterization support. In trainees, CAD EYE reduced adenoma miss rate without increasing ADR in one multicenter tandem study. Movie-based testing showed improved endoscopist detection and characterization, but selected only lesions correctly handled by the system, creating spectrum-selection bias.	FDA 510(k) clearance for Fujifilm EW10-EC02/CAD EYE covers real-time detection of colonic mucosal lesions, including polyps and adenomas, with compatible Fujifilm endoscopy equipment. CE-mark availability has been reported for European use. The FDA detection clearance should not be generalized to all CADx configurations.	Real-time CADe with WLI/LCI; CADx characterization with BLI in supported configurations and markets.	Clinically versatile because detection and characterization are integrated in one ecosystem. Evidence is promising but heterogeneous: some studies show reduced miss rate rather than higher ADR, and selected-video designs may overestimate accuracy. Generalizability outside Fujifilm equipment is limited by ecosystem dependence.
**ENDO-AID (OIP-1)** [77,78]	**Olympus**	Developed using approximately 12 million images and videos from Japan and other countries. The reviewed report does not specify patient count, center distribution, lesion-class balance, demographic composition, or the exact train/validation split. A Japanese evaluation using 185 videos reported high lesion-level sensitivity.	Randomized evaluation among endoscopists-in-training showed higher ADR and adenomas per colonoscopy, particularly for small and nonpedunculated adenomas. It also showed more non-neoplastic resections and a measurable false-positive burden. Independent evidence in expert and broader real-world populations remains less extensive.	ENDO-AID CADe is marketed by Olympus as a computer-aided detection application for suggesting the potential presence of colonic lesions in compatible Olympus systems, with European availability documented by Olympus materials. US FDA clearance for OIP-1/ENDO-AID was not established in the reviewed materials, and separate Olympus/Odin Vision products should not be conflated with ENDO-AID.	Real-time CADe integrated with compatible Olympus EVIS X1 processors, principally during withdrawal.	The unusually large stated development corpus is a strength, but inadequate reporting of patient-level composition prevents assessment of representativeness and leakage risk. The trainee trial supports educational utility, while increased non-neoplastic resection highlights that detection gains do not automatically equal net clinical benefit.
**EndoBRAIN/EndoBRAIN-EYE** [67,68]	**Japanese academic-industrial collaboration; Cybernet Systems/Olympus-linked endocytoscopy ecosystem**	EndoBRAIN development is based mainly on Japanese high-magnification endocytoscopy and image-enhanced datasets, with histopathology as reference. Reviewed publications report prospective validation in hundreds of patients/lesions for optical diagnosis, but complete proprietary training-set composition and external demographic diversity are not consistently available.	Prospective clinical studies and international image-based comparisons report high CADx performance for diminutive neoplastic versus non-neoplastic lesions. Much of the evidence is modality-specific and originates from expert Japanese centers; broad community and cross-platform external validation is limited.	Regulatory approval has been reported in Japan for supported optical-diagnosis applications. FDA clearance and a clearly documented CE scope were not established in the reviewed materials; they should be listed as not verified rather than assumed.	CADx optical characterization, especially with endocytoscopy or magnifying image-enhanced endoscopy; EndoBRAIN-EYE adds lesion-detection support.	Technically mature for a narrow, highly standardized imaging context. High accuracy may not transfer to routine non-magnified colonoscopy, other endoscope ecosystems, or lower-prevalence settings. It should not be compared directly with general-purpose CADe systems without acknowledging the different task and input modality.
**DISCOVERY** [60,79]	**PENTAX Medical**	Uses a deep neural network for real-time CADe. The reviewed multicenter trial did not disclose the size, patient composition, lesion spectrum, annotation process, or geographic diversity of the proprietary training dataset. An unpublished retrospective analysis cited in the trial indicated approximately 11% SSL representation among training/evaluation lesions.	International randomized evaluation across university and non-university centers in Europe and Canada found no significant ADR or APC improvement among experienced endoscopists, but found more SSLs per colonoscopy. Validation populations were not used for system development, reducing overfitting concerns.	A CE mark has been reported for PENTAX Medical DISCOVERY for AI-assisted real-time polyp detection during colorectal examination. FDA clearance was not established in the reviewed regulatory materials. The system should be described as CADe with compatible PENTAX systems, not as CADx.	Real-time CADe with compatible PENTAX high-definition colonoscopy systems.	Independent multicountry validation is a major strength and provides an important neutral result. Lack of ADR improvement illustrates that platform benefit is context-dependent and may be attenuated by high baseline ADR. The SSL signal is clinically interesting but was secondary and requires confirmation.
**Eagle-Eye 5.1** [78,79]	**Xiamen Innovision, China**	YOLOv3/Darknet-53 model trained on 112,199 WLI still images from one hospital: 64,134 images with histopathologically confirmed polyps and 48,065 without polyps; 90:10 internal split. Fine-tuned with 21 videos from Olympus and Fujifilm systems and evaluated on an independent 40,000-image dataset. Dedicated SSL enrichment was absent.	Large six-center randomized screening trial in 3059 asymptomatic participants showed higher overall ADR, advanced ADR, and APC with AI. Validation was geographically multicenter, but remained predominantly Chinese and used a single software platform. SSL detection did not improve.	FDA clearance and CE marking were not established in the reviewed sources. The platform should be described as investigational or locally deployed unless jurisdiction-specific authorization is documented.	Real-time CADe for colorectal polyp detection across several compatible endoscopy systems.	Among the most transparently reported training datasets in the reviewed literature and supported by a large multicenter RCT. Limitations include single-center training data, uncertain demographic diversity, no dedicated SSL training, and limited evidence outside China. Regulatory maturity appears below that of established Western commercial systems.
**EndoAngel/EndoScreener** [78,79,80]	**Wuhan Endo Angel Medical Technology and collaborating academic centers**	The reviewed manuscripts describe deep-learning CADe and quality-control development using large colonoscopy image/video collections, but the exact dataset differs by version and is not consistently disclosed. Published systems may combine lesion detection with withdrawal-speed, blind-spot, and bowel-preparation monitoring; version-specific training data must therefore be reported.	Multiple Chinese randomized studies report improved ADR/PDR or procedural quality. Generalizability outside Asian centers and across software versions is less certain. The included 2021 multicenter system study used 117,048 WLI video frames from six centers, but it was a separately developed RetinaNet system and should not automatically be labeled EndoAngel.	EndoScreener has FDA 510 (k) clearance as a gastrointestinal lesion software detection system for real-time CADe of suspected colorectal polyps. CE status and regulatory clearance of EndoAngel quality-assessment functions were not verified in the reviewed materials. EndoScreener and EndoAngel versions should not be treated as interchangeable.	Real-time CADe; selected EndoAngel configurations also provide computer-aided quality assessment and training support.	Clinical evidence is substantial but nomenclature and versioning are major risks for synthesis. Studies frequently evaluate research or locally configured versions, making regulatory and performance claims difficult to transfer to the marketed product. Clear separation of CADe and CAQ functions is essential.
**SKOUT** [79,80]	**Iterative Scopes/** **Iterative Health**	Proprietary training-data characteristics were not reported in the reviewed manuscripts. Publicly available regulatory summaries identify SKOUT as an AI-based real-time CADe system, but detailed patient numbers, lesion distribution, imaging systems, and demographic diversity require dedicated technical documentation.	Independent platform-specific validation was not available in the reviewed evidence set. Therefore, numerical performance comparisons with GI Genius, CAD EYE, ENDO-AID, or DISCOVERY should not be made without dedicated SKOUT studies.	SKOUT has FDA 510 (k) clearance for real-time detection of potential colorectal polyps during white-light colonoscopy in adults undergoing screening or surveillance. It assists trained gastroenterologists and does not replace clinical judgment. Verified CE status was not established in the reviewed materials.	Real-time CADe as an adjunct during colonoscopy.	Regulatory availability is clearer than the clinical evidence base identified in this review. Without full training-data disclosure and independent head-to-head validation, comparative claims should remain conservative.

Abbreviations: ADR, adenoma detection rate; APC, adenomas per colonoscopy; CADe, computer-aided detection; CADx, computer-aided diagnosis; CAQ, computer-aided quality assessment; FDA, U.S. Food and Drug Administration; SSL, sessile serrated lesion; WLI, white-light imaging; LCI, linked-color imaging; BLI, blue-light/blue-laser imaging.

## Data Availability

This manuscript is a review article and does not include original research data collected by the authors. The content of the paper is based on the analysis, synthesis and critical evaluation of the results of previously published research. All data, information and materials used in the preparation of the manuscript originate from publicly available and adequately referenced sources. All data presented in this article are contained within the article itself. Appropriate references are listed in the paper, which enables verification and access to the sources used. Given the nature and methodology of this paper, a separate statement on the availability of original data is not applicable.
